# Genetic and Molecular Characterization of Avian Influenza A(H9N2) Viruses from Live Bird Markets (LBM) in Senegal

**DOI:** 10.3390/v17010073

**Published:** 2025-01-08

**Authors:** Mamadou Malado Jallow, Moussa Moise Diagne, Marie Henriette Dior Ndione, Mamadou Aliou Barry, Ndiendé Koba Ndiaye, Davy Evrard Kiori, Marie Pedapa Mendy, Déborah Goudiaby, Gamou Fall, Malick Fall, Ndongo Dia

**Affiliations:** 1 Département de Virologie, Institut Pasteur de Dakar, Dakar BP 220, Senegal; mamadoumalado.jallow@pasteur.sn (M.M.J.); moussamoise.diagne@pasteur.sn (M.M.D.); marie.ndione@pasteur.sn (M.H.D.N.) ; ndiendekoban@gmail.com (N.K.N.); davy.kiori@pasteur.sn (D.E.K.); mendymary15@gmail.com (M.P.M.); deborah.goudiaby@pasteur.sn (D.G.) ; gamou.fall@pasteur.sn (G.F.); 2 Département de Biologie Animale, Faculté des Sciences et Techniques, Université Cheikh Anta DIOP de Dakar, Dakar BP 206, Senegal; malickfal@yahoo.fr; 3 Unité d’Epidémiologie des Maladies Infectieuses, Institut Pasteur de Dakar, Dakar BP 220, Senegal; aliou.barry@pasteur.sn

**Keywords:** avian influenza viruses, H9N2, genetic, live bird markets, surveillance, Senegal

## Abstract

Despite extensive experience with influenza surveillance in humans in Senegal, there is limited knowledge about the actual situation and genetic diversity of avian influenza viruses (AIVs) circulating in the country, hindering control measures and pandemic risk assessment. Therefore, as part of the “One Health” approach to influenza surveillance, we conducted active AIV surveillance in two live bird markets (LBMs) in Dakar to better understand the dynamics and diversity of influenza viruses in Senegal, obtain genetic profiles of circulating AIVs, and assess the risk of emergence of novel strains and their transmission to humans. Cloacal swabs from poultry and environmental samples collected weekly from the two LBMs were screened by RT-qPCR for H5, H7, and H9 AIVs. Subsequently, a subset of H9-positive samples was selected for whole sequencing. From December 2023 to October 2024, 499 samples were tested, and AIV was detected in 58.3% of them. Among these, A/H9N2 was the only subtype detected in both markets, with a detection rate of 47.7% (82/172) in Thiaroye and 35.3% (42/119) in Tilene, resulting in an overall positivity rate of 42.6% (124/291). Genome sequencing of 22 A/H9N2 isolates, including 11 poultry drinking water samples, 7 carcass wash water samples, 3 fecal samples, and 1 cloacal swab, yielded 7 complete and 15 partial genomic sequences. Phylogenetic analyses of the resulting sequences showed that the A/H9N2 isolates obtained in this study formed a monophyletic cluster and were closely related to the Senegalese human strain (A/Senegal/0243/2019) identified through the national influenza sentinel surveillance program. These strains were also closely related to the A/H9N2 viruses of the G1 lineage circulating in neighboring countries, suggesting cross-border transmission. The A/H9N2 strains carried the low pathogenicity RSSR/GLF motif at the HA cleavage site and possessed several key amino acid mutations, including HA-I155T and HA-Q226L, which are associated with human host adaptation, PB2-T105V, PB2-A661T, and PB2-A588V, which are linked to the human-to-human transmission and increased polymerase activity, NS2-T14M, NS2-M100I, NS1-I106M, NS1-V222M, NS1-E223A, NS1-I226V, NS1-E227G, and NS1-P228S, which are known to alter virulence (increased or reduced) in humans or mice, and M2-S31N, which promotes drug resistance. Seven potential N-glycosylation sites were predicted in the HA protein and six in the NA protein. The selection pressure analysis revealed that the A/H9N2 isolates were primarily under neutral evolution or purifying selection pressure. Overall, our findings highlight the potential for cross-species transmission of Senegalese A/H9N2 viruses, emphasizing the need for sustained monitoring of these viruses in both animal and human populations.

## 1. Introduction

Among respiratory viruses, influenza represents the greatest public health threat in terms of both seasonal and potential pandemic disease [1]. It is caused by viruses belonging to the *Orthomyxoviridae* family, which consists of segmented, single-stranded, negative-sense RNA viruses classified into four types: influenza A, B, C, and D [2]. Influenza A viruses (IAVs) are commonly characterized by their combinations of surface glycoproteins, hemagglutinin (HA) and neuraminidase (NA), giving rise to a multitude of different subtypes [3,4]. To date, 19 HA (H1–H19) and 11 NA (N1–N11) subtypes of IAVs have been recognized [5,6]. Of these, subtypes H1-H16 and H19 (recently identified in wild birds as a potential new subtype) and N1-N9 have primarily been detected in avian species, whereas H17-H18 and N10-N11 have only been found in bats [7,8]. Wild aquatic birds, which harbor almost every known subtype of influenza, are the primary natural reservoir of IAV. These viruses sporadically spill over from wild bird hosts to infect domestic poultry [9].

Avian influenza virus (AIV) of the A/H9N2 subtype has gained increasing global attention in recent years due to its extensive circulation in poultry populations and its sporadic zoonotic transmission to humans. Since 2003, nearly 130 human infections with A(H9N2) have been reported, predominantly in individuals with close contact with poultry or contaminated environments, such as live bird markets. In Africa, six human cases of A(H9N2) infection have been reported so far: four in Egypt, one in Senegal, and one in Ghana [10,11]. Most importantly, A/H9N2 AIVs have the potential to act as a genetic donor for other influenza viruses, contributing gene segments that can reassort with other AIV subtypes, such as A/H7N9, which has caused more than 1500 human cases and 600 deaths in China, and A/H5N1, responsible for over 800 cases and 400 deaths worldwide since its emergence in 1996 [12].

In low- and middle-income countries, including Senegal, backyard poultry farming is an important livelihood activity for many people living in rural, urban, and semi-urban areas. Live bird markets (LBMs) play a significant role in the poultry marketing system in these regions. The presence of various species of domestic birds from different regions, close and frequent interactions between poultry and humans, and poor sanitary conditions create an ideal environment in LBMs for the reassortment and emergence of influenza viruses with pandemic potential [13]. Therefore, active surveillance in LBMs is crucial for understanding the genetic characteristics of the circulating AIV and preventing their spread to humans.

In Senegal, despite reporting the first human case of avian influenza A/H9N2 infection [11] and experiencing a large outbreak of HPAIV A(H5N1) in poultry farms and among white pelicans, which suffered high mortality rates in 2020 [14], the country lacks adequate surveillance of influenza in animals (poultry, pigs, wild birds, etc.). This highlights the weaknesses in influenza surveillance, even though the country has a long history of monitoring influenza in humans [15]. So, here, we conducted active surveillance of AIV in two LBMs in Dakar to better understand the dynamics and diversity of influenza viruses in Senegal, obtain genetic profiles of circulating AIVs, and assess the risk of emergence of novel strains and their transmission to humans.

## 2. Materials and Methods

### 2.1. Study Design and Sample Collection

As part of the “One Health” approach to influenza surveillance, the Institut Pasteur de Dakar (IPD), through the National Influenza Center (NIC), initiated active AIV surveillance in December 2023 at two LBMs in Dakar, the capital city of Senegal, Thiaroye, a large LBM located in the suburbs of Dakar, and Tilene, a smaller LBM located in central Dakar. Environmental and poultry samples, including cloacal swabs from birds, carcass wash water (large buckets of water used to wash freshly slaughtered poultry), poultry feces, and poultry drinking water, were collected weekly. During market visits, poultry were also purchased from vendors, and blood samples were collected in 15 mL Falcon sterile tubes immediately after culling. Specimens were collected in sterile tubes containing 2 mL of universal viral transport medium (VTM; Becton Dickinson and Company, Milan, Italy) and transported the same day at a controlled temperature (4 °C) to the NIC located to IPD. Upon arrival in the laboratory, specimens were immediately processed for influenza A screening and identification. Aliquots of each sample were also stored at −80 °C for biobanking or additional analysis.

**Figure 1:**
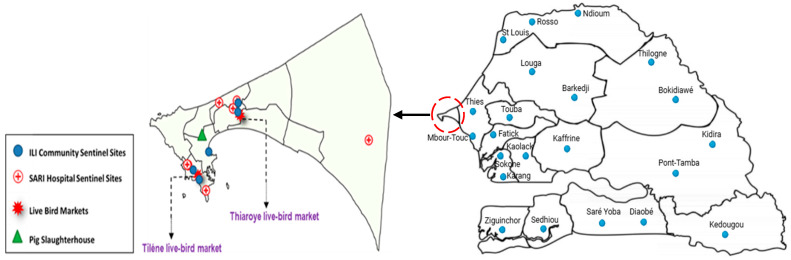
Live-bird market locations in Dakar (orange shadowed dots) and sentinel sites involved in the surveillance of human zoonotic influenza infections.

Surveillance for human zoonotic influenza infections is conducted through a country-wide influenza-like-illness (ILI) sentinel surveillance system, which includes 30 community sentinel sites distributed across the 14 administrative regions of Senegal, and hospital-based surveillance of severe acute respiratory illness (SARI) in 7 referral hospitals in Dakar (Figure 1).

### 2.2. Detection of AIVs Using RT-qPCR

The QIAamp Viral RNA Kit (QIAGEN, Valencia, CA, USA) was used to extract viral RNA from 200 μL of VTM fluid containing poultry samples according to the manufacturer’s instructions. Extracted RNA was subjected to real-time reverse transcription-polymerase chain reaction (rRT-PCR) to initially screen for all IAVs, using specific primers and a probe targeting a conserved region of the influenza matrix gene. Samples that tested positive for influenza A (Ct-values < 37) were subsequently subtyped via rRT-PCR assays using primers and a probe that specifically amplified and discriminated the hemagglutinin (HA) and neuraminidase (NA) genes of H9, H5, and H7 avian influenza subtypes. For the detection of H5 and H7 subtypes, primers and probes were sourced from the Centers for Disease Control and Prevention (CDC) International Reagent Resource (IRR; https://www.internationalreagentresource.org/Home.aspx, accessed on 10 November 2023), whereas a set of primers and a probe previously published was used for the detection of the A/H9N2 virus [16]. The AgPath-ID™ One-Step RT-PCR kit (Ambion, Foster City, CA, USA) was used for real-time amplification in an Applied Biosystems QuantStudio 5 Real-Time PCR device (Thermo Fisher Scientific, Inc., Waltham, MA, USA) using standard protocols. Briefly, for each sample (for both initial screening and subtyping), real-time PCR was carried out in a total reaction volume of 25 μL consisting of 5 μL of nuclease-free water, 0.5 μL of each primer, 0.5 μL of the probe, 12.5 μL of 2X RT-PCR Buffer, 1 μL of 25X RT-PCR Enzyme Mix, and 5 μL of RNA template under the following cycling conditions: reverse transcription step of 15 min at 50 °C, initial denaturation step of 3 min at 95 °C, followed by 40 PCR cycles of 15 s at 95 °C and 30 s at 55 °C. Appropriate negative and positive control specimens were run alongside each reaction for validation. When all controls met the stated requirements, any sample with a cycle threshold (Ct) below 37 was deemed positive.

### 2.3. Genome Amplification and NGS Sequencing of AIV

A subset of 22 H9-positive samples (15 samples from Thiaroye LBM and 7 from Tilene LBM), including 11 poultry drinking water samples, 7 carcass wash water samples, 3 fecal samples, and 1 cloacal swab, were selected to undergo full-genome sequencing on an Illumina sequencing platform (Illumina, San Diego, CA, USA).

#### 2.3.1. cDNA Synthesis

For cDNA synthesis, viral RNA was extracted from selected AIV-positive samples, and reverse transcription was carried out using RevertAid First Strand cDNA Synthesis Kit (Thermo Scientific, Vilnius, Lithuania) and the universal primer Uni12 (AGCAAAAGCAGG). Briefly, for each sample, a mixture of 15 μL of RNA and 2 μL of Uni12 (10 μM) primer was initially incubated at 65 °C for 5 min and immediately placed on ice for 1 min. Subsequently, the following components were added to each tube: 6 μL of 5× Reaction Buffer, 1.5 μL of RiboLock RNase Inhibitor (RI), 1.5 μL of RevertAid Reverse Transcriptase (RT), and 3 μL of dNTP Mix (10 μM). The reaction was performed at 42 °C for 1 h and was terminated by heating at 70 °C for 5 min.

#### 2.3.2. Multi-Segment PCR of AIV

To characterize AIV by next-generation sequencing, complete genomes (all eight-gene segments) were amplified through a single conventional PCR run by using the LongAmp Tag 2X Master Mix kit (New England Biolabs, Ipswich MA, USA) with primers MBTuni-12 (5′-ACGCGTGATCAGCAAAAGCAGG-3′) and MBTuni-13 (5′-ACGCGTGATCAGTAGAAACAAGG-3′) previously reported by Zhou et al. [17]. Briefly, for each sample, PCR amplification was carried out in a total reaction volume of 50 μL consisting of 11 μL of nuclease-free water, 2 μL of each primer (diluted at 10 μM), 25 μL of Master mix and 10 μL of cDNA template. The thermal cycling conditions were as follows: an initial denaturation at 94 °C for 30 s, five cycles of 94 °C for 30 s, 45 °C for 30 s, and 65 °C for 3 min, followed by 45 cycles of 94 °C for 30 s, 56 °C for 30 s, and 65 °C for 3 min, and a final extension at 65 °C for 10 min.

PCR products were analyzed on a 1% agarose gel stained with ethidium bromide, using 1×TAE as the electrophoresis running buffer. Bands containing amplicons were excised and purified with the NucleoSpin^®^ Gel and PCR Clean-Up kit (Macherey-Nagel GmbH & Co. KG, Düren, Germany) according to the supplier’s protocol. After purification, the amplicons were quantified and normalized to approximately 100 ng using the Qubit fluorometer (Invitrogen Life Technologies) before proceeding to NGS sequencing.

#### 2.3.3. Next Generation Sequencing

For whole-genome sequencing of AIVs, an amplicon-based next-generation sequencing approach was used. Briefly, the pooled PCR products underwent bead-based tagmentation using the Nextera DNA Flex library preparation kit. The adapter-tagged amplicons were cleaned up using AMPure XP purification beads (Beckman Coulter, High Wycombe, UK) and amplified through one round of PCR. The PCR products were indexed using the Nextera CD indexes (Illumina, Inc.) according to the manufacturer’s instructions. The resulting libraries were quantified using a Qubit 4.0 fluorometer (Invitrogen Inc., Waltham, MA, USA) with the Qubit dsDNA High Sensitivity assay, following the manufacturer’s protocol. The pooled libraries were normalized to a 1 nM concentration, and 5 μL of each normalized pool containing unique index adapter sets was combined into a new tube. The final library pool was denatured with 0.1 N sodium hydroxide, and a 12 pM sample library was spiked with 10% PhiX. Libraries were loaded onto an Illumina MiSeq platform with 151 bp paired-end reads using the Miseq Reagents kit v3, following the manufacturer’s protocol (Illumina, San Diego, CA, USA).

#### 2.3.4. AIV Genome Assembly

To generate the consensus genomes of AIVs in all samples, raw sequencing data in FASTQ files were processed and analyzed using the Chan-Zuckerberg ID (CZ ID), a cloud-based open-source bioinformatics pipeline for metagenomics sequencing data (https://czid.org/, accessed on 26 April 2024). Briefly, raw FASQ files were uploaded to the CZ ID portal through the web Application. Adapter sequences were trimmed, and then host and duplicate sequences were filtered. The remaining short-read sequences were aligned to the NCBI nucleotide (nt) and nonredundant protein (nr) databases using GSNAP and Rapsearch2, respectively. Putative accessions were assigned to each read using the NCBI accession2taxid database and a BLAST+ (v 2.6.0) database. In parallel, short reads were de novo assembled into contigs using SPADES. Raw reads were mapped back to the resulting contigs using Bowtie2 to identify the contig to which each raw read belongs. Finally, each contig was aligned to the set of possible accessions represented by the BLAST database to improve the specificity of alignments to all the underlying reads. Consensus genomes were generated using bcftools consensus, incorporating variants with a minimum depth of coverage of 10 and a consensus allele frequency of 0.50 [18].

### 2.4. Phylogenetic Analysis

To investigate the genetic relationships, the newly generated consensus genomes in this study were combined with complete genome sequences of A/H9N2 viruses, including reference sequences of known genotypes, which were downloaded from the NCBI (https://www.ncbi.nlm.nih.gov/) and GISAID (https://gisaid.org/) databases. The eight datasets corresponding to the eight gene segments of A/H9N2 were aligned using MAFFT implementing the FFT-NS-2 algorithm [19] and then manually edited and trimmed using BioEdit v7.1.3.0.

Maximum likelihood (ML) phylogenetic trees for each gene segment were inferred using IQ-Tree (v2.0.3) with the best-fit nucleotide substitution model (GTR + I + G4) [20] and visualized using FigTreev1.4.4 (http://tree.bio.ed.ac.uk/software/figtree, accessed on 27 May 2024). The robustness of the tree topology was estimated with 1000 ultrafast bootstrap (UFBoot) replicates, and bootstrap values ≥ 70% were considered statistically significant.

### 2.5. Genetic Analysis of Amino Acid Residues of A/H9N2 Viruses

Deduced amino acids of the individual sequences were analyzed using the GISAID FluServer (https://flusurver.bii.a-star.edu.sg/, accessed on 27 May 2024) to identify reported molecular markers that may be involved in increased virus virulence, mammalian transmission, receptor-binding specificity, and antiviral drug resistance.

Potential N-linked glycosylation sites were predicted for HA and NA using the NetNGlyc1.0 web Server (https://services.healthtech.dtu.dk/services/NetNGlyc-1.0/, accessed on 27 May 2024) with default parameters. Only threshold values greater than 0.5 for the average potential score were considered potential glycosylation sites.

### 2.6. Selection Pressure Analysis

To evaluate the site-specific selection pressures acting on each gene segment of A/H9N2 from Senegal, we estimated the ratio (ω) of non-synonymous (dN) to synonymous (dS) mutations (ω = dN/dS) using the HyPhy software package accessed via the Datamonkey webserver (https://www.datamonkey.org/, accessed on 27 May 2024). Four different methods were used, including Fixed Effects Likelihood (FEL), Single Likelihood Ancestor Counting (SLAC), Mixed Effects Model of Evolution (MEME), and Fast Unconstrained Bayesian AppRoximation (FUBAR). An ω < 1 indicates negative or purifying selection pressure; ω = 1 implies neutral evolution; and ω > 1 indicates positive selection. For the SLAC, FEL, and MEME methods, sites with *p*-values < 0.1 were accepted as candidates for selection, whereas for FUBAR, a posterior probability > 0.9 was considered significant.

### 2.7. Data Management and Statistical Analysis

All data obtained from this active AIV surveillance, including the laboratory results, were recorded in a standard Excel spreadsheet. Subsequent data analysis was carried out using RStudio version 2024.04.1-748 software (RStudio Inc., Boston, MA, USA).

### 2.8. Ethical Statement and Permission

Permission was sought from poultry vendors and workers at the live-bird markets for sample collection. All procedures performed in studies involving animal samples adhered to international, national, and institutional guidelines for the care and use of animals.

## 3. Results

### 3.1. Sample Collection and Detection of AIV

From December 2023 through October 2024, we collected and analyzed 499 samples from the two live bird markets. Of these, 283 (56.7%) samples were collected from the Thiaroye LBM and 216 (43.3%) from the Tilene LBM. The collected samples included 139 (27.8%) cloacal swabs from clinically healthy birds (mainly from layers, broilers, and pigeons), 128 (25.6%) poultry feces, 138 (27.6%) poultry drinking water, and 94 (18.8%) carcass wash water.

Avian influenza virus (AIV) was detected in 58.3% (291/499) of the overall samples tested. Of these positive cases, 59.1% (172/291) were from Thiaroye and 40.9% (119/291) from Tilene. AIV was most frequently detected in carcass wash water and poultry drinking water, accounting for 28.9% (84/291) and 27.8% (81/291) of the overall positive cases, respectively. In feces and cloacal swab samples, the virus was detected in 22.3% (65/291) and 21% (61/291) of the cases, respectively.

Among the tested AIV subtypes (H5, H7, and H9), the H9 subtype was the only virus detected in both markets, with a detection rate of 47.7% (82/172) in Thiaroye and 35.3% (42/119) in Tilene, resulting in an overall positivity rate of 42.6% (124/291) (Table 1).

### 3.2. Sequencing and Phylogenetic Analysis

After molecular identification of AIV, 7 complete genomes and 15 partial genomes (7 gene segments) were successfully obtained from the 22 H9 viruses with relatively high viral loads (Ct value < 30) that were attempted for NGS sequencing. Table 2 provides a detailed description of the complete genomes of the A/H9N2 viruses analyzed in this study, including coverage and sequencing depth for each gene segment.

We preliminarily analyzed all the sequences generated in this study by performing a nucleotide query in the Genbank database to identify sequence matching of each gene segment. The BLAST results confirmed all eight segments as belonging to the A/H9N2 AIV subtype. Subsequently, a phylogenetic analysis was performed to better understand the genetic evolution of the A/H9N2 AIVs isolated from LBMs.

**Figure 2:**
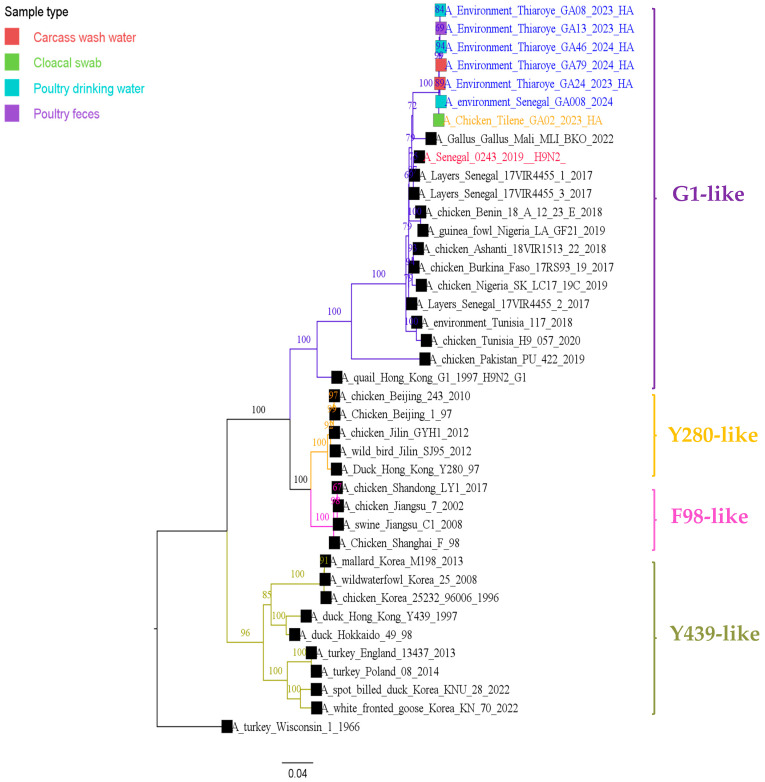
Maximum-likelihood phylogenetic tree based on complete nucleotide sequences of the HA genes of Senegalese A/H9N2 viruses isolated from live bird markets (LBM). The tree was generated using IQ-TREE (v2.0.6) and visualized with FigTree (v1.4.4). Statistical significance was assessed using 1000 bootstrap replicates, and the best-fit model was determined by the software. Sequences from Senegal are highlighted in blue for isolates obtained from the Thiaroye LBM, in orange for isolates from the Tilene LBM, and in red for the human A/H9N2 isolate.

**Figure 3:**
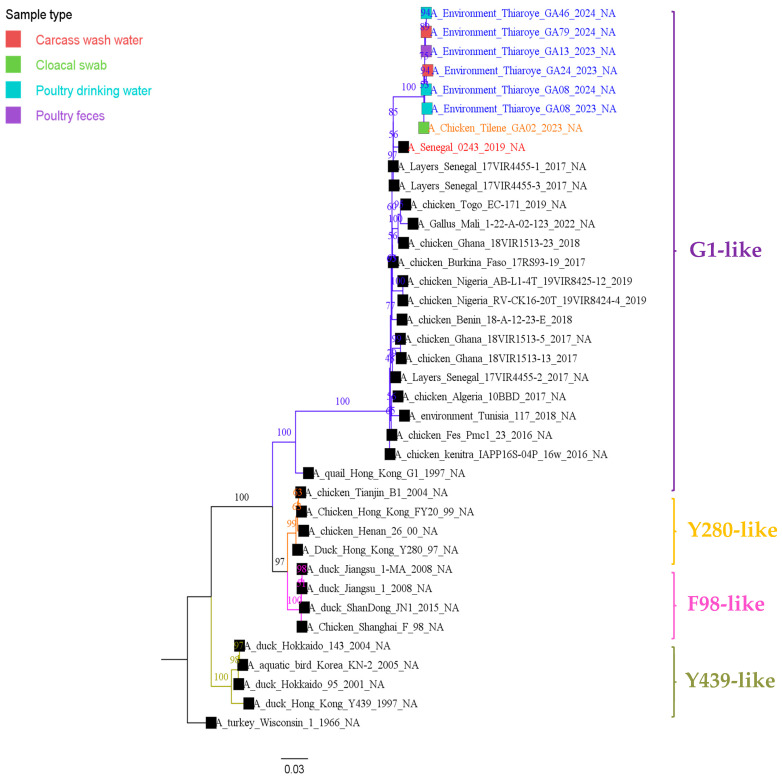
Maximum-likelihood phylogenetic tree based on complete nucleotide sequences of the NA genes of Senegalese A/H9N2 viruses isolated from live bird markets (LBM). The tree was generated using IQ-TREE (v2.0.6) and visualized with FigTree (v1.4.4). Statistical significance was assessed using 1000 bootstrap replicates, and the best-fit model was determined by the software. Sequences from Senegal are highlighted in blue for isolates obtained from the Thiaroye LBM, in orange for isolates from the Tilene LBM, and in red for the human A/H9N2 isolate.

As shown in Figure 2 and Figure 3, the maximum likelihood phylogenetic analysis based on the complete surface genes (HA and NA) showed that the A/H9N2 isolates of this study belonged to the G1(A/quail/Hong Kong/G1/1997) lineage, and clustered with A/H9N2 viruses identified in West and North African chickens and wild birds between 2016 and 2022. The nucleotide similarity ranged between 93.83% and 96.55% for the HA and 93.62% and 96.77% for the NA. These isolates were also closely related to the A/H9N2 human case (A/Senegal/0243/2019) identified in 2019 in Senegal through the national influenza sentinel surveillance. The phylogenetic trees of the internal gene segments (PB2, PB1, PA, NP, M, and NS) reflect the same topology as the HA and NA phylogeny (Supplementary Materials Figures S1–S6).

### 3.3. Genetic Analysis of Amino Acid Residues of A/H9N2 Strains

To gain further insights and assess the zoonotic potential posed by the avian A/H9N2 viruses from this study, we investigated the molecular signatures associated with the disease phenotype, such as the human host adaptation markers, receptor-binding specificity, increased virulence, and susceptibility to antiviral drugs. FluServer (https://flusurver.bii.a-star.edu.sg, accessed on 27 May 2024) was used to screen the Senegalese A/H9N2 viruses for mutations of interest. The analysis revealed that the A/H9N2 isolates from this study exhibited typical contemporary poultry A/H9N2 virus features. All the isolates shared a similar amino acid motif of RSSR/GLF at the cleavage sites between the subunits HA1 and HA2 of the HA protein (residues 335–341, H9 numbering), which is a typical characteristic of low pathogenicity AIVs for chickens [10]. The receptor binding sites (RBSs) of the HA protein harbored several amino acid substitutions that promote preferential binding to human-like α2-6-linked sialic acid (SA α2-6) receptors, including well-known residues I155T and Q226L (H3 numbering). In addition, the HA proteins of these viruses contained numerous other amino acid mutations, including S121T, S143T, S145T, A150S, N167G, A168N, N191H, Q235I, V327I, and S353P, which are associated with host specificity shift or increased virulence in mammalian species (Table 3). Molecular markers, such as T105V and A661T, involved in adaptation to human-to-human transmission were identified in the PB2 genes of 100% of the isolates [21]. The A588V mutation, which is associated with increased polymerase activity of AIVs in MDCK (Madin-Darby Canine Kidney), HEK293T (Human Embryonic Kidney 293T), and avian DF-1 cell lines, as well as higher virulence in mice, was also encountered in PB2 [22]. However, well-known substitutions in PB2, namely E627K and D701N, were not detected. Amino acid substitutions H99Y, V336X, K207X, and N375X in PB1, V100I, and S409N in PA, and E372D, R416X, K184X, W120X, and R267X in NP, which are associated with either host specificity shift from avian to human or increased virulence in chicken, were found in some isolates. Only a small number of mutations in M1 and NS2 proteins that increase AIV virulence in mice or avian models were detected. In the M1 protein, T139N, which is associated with increased virulence in mice, was the only molecular marker of concern identified; in NS2, T14M and M100I, which are associated with reduced virulence in humans and mice, were the mutations identified. In contrast to the NS2 protein, several amino acid substitutions, including E70A, I81T, I106M, S212P, V222M, E223A, I226V, E227G, and P228S, were detected in the NS1 protein when compared with the reference strain A/Quail/Hong Kong/G1/1997. These substitutions are associated with changes in host specificity (avian-to-human adaptation) or alterations in virulence (increased or reduced) in humans or mice.

### 3.4. Antiviral Resistance Mutational Analysis

Molecular analysis of the M2 protein showed that all the Senegalese A/H9N2 viruses contained the primary adamantine-resistance marker, S31N. In the N2 protein, the H274Y amino acid substitution, which is the NA mutation most frequently associated with oseltamivir resistance [41], was not detected in any of the Senegalese strains. However, compared to the reference virus A/Chicken/Hong Kong/G9/1997, several mutations reported to be involved in strong or mild resistance to drugs, such as oseltamivir, zanamivir, and laninamivir, were observed. These mutations included E141D, K38Q, E41K, A149T, T153S, A170G, A263V, R331T, N358S, I392T, K432R, and F466L. None of the Senegalese isolates harbored the PA-I38T mutation associated with resistance to the recently licensed cap-dependent endonuclease inhibitor, baloxavir marboxil [42].

### 3.5. Prediction of Potential N-Glycosylation Sites on the HA and NA Genes and Selection Pressure Analysis

Using the NetNGlyc 1.0 server, after scanning the HA and NA glycoproteins, six potential N-glycosylation sites were predicted in the HA1 subunit at amino acid positions ^29^NSTE^32^, ^82^NPSC^85^, ^105^NGTC^108^, ^141^NVTY^144^, ^298^NSTM^301^, and ^305^NISK^308^, and one potential N-glycosylation site was predicted in the HA2 subunit at position ^492^NGTY^495^ (Table 4). For the NA protein, six conserved N-glycosylation sites were predicted at positions ^44^NISN^47^, ^61^NKTE^64^, ^70^NITI^73^, ^146^NGTT^149^, ^234^NGTC^237^, and ^329^NDTS^332^. The amino acid change from asparagine (N) to aspartic acid (D) at position 218 in the HA protein led to the loss of a potential N-glycosylation site. However, the mutation HA M107T (H9 numbering) creates a new potential N-glycosylation site at position 105. In the NA protein, mutations T71I, N86I, N309D, and N402D led to the loss of the related potential N-glycosylation sites in most of the isolates, while mutations S70N and D329N created new potential N-glycosylation sites at positions 70 and 329, respectively.

Furthermore, the selection pressures acting on each gene segment of the Senegalese A/H9N2 AIV were estimated by assessing the ratio of non-synonymous to synonymous substitutions (dN/dS). Based on the results obtained using the Datamonkey webserver, sites in each RNA gene segment were mainly under neutral evolution or negative selection pressure. Only one codon at position 183 in the HA gene was found to be under diversifying positive selection with the FUBAR model.

## 4. Discussion

In recent years, the circulation of various AIV subtypes in poultry from LBMs, particularly H5 and H7, has led to genetic exchanges among A/H9N2, A/H7N9, and A/H5N1, resulting in significant outbreaks in humans with high mortality rates [13,43,44]. However, despite being recognized as a donor of internal genes to other AIV subtypes, far less attention has been given to the pandemic potential of A/H9N2 viruses, probably because of its low pathogenicity in poultry and the mild disease they cause in humans. Senegal faces a heightened risk for the emergence of new AIV strains due to the predominant practice of rearing domestic birds in traditional farming systems with insufficient biosecurity measures. This risk is coupled with the presence of the Djoudj National Bird Park (a UNESCO World Heritage site), one of the largest bird sanctuaries in the world, which acts as a stopover for millions of wild aquatic birds each year [14], providing excellent grounds for reassortment events between AIVs. Other wetlands scattered throughout the country also serve as bird sanctuaries. While the epidemiology of influenza in humans is well-documented in Senegal [45,46,47], our understanding of the real situation and the genetic diversity of AIVs circulating in poultry is largely unknown, hindering control measures and pandemic risk assessment. To fill this knowledge gap, we conducted active surveillance of AIVs in two LBMs in Dakar to better understand the dynamics and diversity of AIVs and to assess the risk of the emergence of novel strains and their potential transmission to humans.

During the eleven months of AIV surveillance (December 2023 through October 2024), 58.3% of the 499 samples collected from the two LBMs tested positive for AIV. This rate is relatively similar to that reported in a longitudinal surveillance of two LBMs in Cambodia, with authors reporting an AIV detection rate of 55% [48]. The reasons for the high detection rate in these LBMs may include various management practices and environmental factors, such as poor biosecurity measures and high poultry density. The overall prevalence reported in this study is higher compared to those reported in previous studies from several countries, including Mali with 16% [49], Bangladesh with 23% [50], and Hubei Province (China) with 26.6% [8]. The variations in AIV detection rates in different areas can be explained by several factors, including the type of samples collected (tracheal swabs, cloacal swabs, fecal samples, or environmental samples might yield different detection rates) and the local bird populations. For instance, different regions may have varying bird populations, including differences in species diversity and density, which can influence AIV prevalence. Unlike several reports from Asia where co-circulation of H5, H7, and H9 subtypes has been noted in poultry in LBMs [48,51], A/H9N2 was the only avian influenza subtype found in this study, accounting for 42.6% of the overall AIV positive cases. However, the circulation of HPAIV H5N1 has previously been reported in a poultry farm in the Thies administrative region and in great white pelicans in the Djoudj National Bird Sanctuary [14], with a high mortality rate. The concomitant circulation of A/H5N1 in the country may be an alarming circumstance, considering the possibility that a reassortant H9N2-H5N1 strain with negative agricultural and/or public health implications could emerge and spread, as recently reported in Egypt [52], Burkina Faso [53], and India [54]. Therefore, sustained monitoring of markets that contain wild birds and live poultry, as well as timely analysis of virus evolution and gene mutations, is essential. As has been reported by several authors around the world [48,51], carcass wash water and poultry drinking water were most frequently AIV-positive among different sample types, suggesting that these types of samples should be the main target for surveillance and the sites of detection should serve as primary mitigation points to limit the transmission and propagation of AIV and reduce the risk for the emergence of novel viruses in LBMs.

The phylogenetic analysis of our limited dataset of A/H9N2 viruses supports the circulation of the G1 lineage in Senegal, similar to patterns observed in several other sub-Saharan African countries [10,55,56,57]. The G1 lineage has been known to be circulating in Africa since 2016 and has spread to different countries within the continent [58,59]. This contrasts with some settings in Asia, notably Cambodia, where co-circulation of the three main H9-HA lineages (Y439/97, G1/97, and BJ/94) has been reported [60]. Furthermore, the A/H9N2 strains isolated in Senegal showed close genetic clustering with A/H9N2 viruses previously identified in Mali and in other neighboring countries, confirming the transboundary spread of the infections and the need to enhance the efficacy of the control measures at a transnational level.

Molecular analyses of deduced amino acid sequences revealed the presence of a typical LPAIV RSSR/GLF cleavage motif in the HA1-HA2 connecting peptide of the avian A/H9N2 strains from this study, similar to those found in LPAI A/H9N2 viruses circulating in poultry globally [61,62,63]. However, a different pattern was observed at the HA cleavage site of A/H9N2 viruses isolated from humans and birds in India, where multiple basic amino acids, KSKR↓GLF was found, suggesting a trend for greater pathogenicity in these viruses [4]. As observed in most contemporary avian A/H9N2 viruses [29,49], the strains obtained in this study harbored numerous amino acid changes in the HA RBS, including I155T and Q226L (H3 numbering), which are reported to promote preferential binding to human-like α2-6-linked sialic acid (SA α2-6) receptors and enable efficient replication in human respiratory epithelial cells and in the ferret model [64]. In addition, consistent with previous findings in Egypt, the Senegalese A/H9N2 viruses possessed L234 and H191 (H9 numbering) markers, which are associated with a shift in HA preference from avian α-2,3 sialic acid (SA) receptors to human α-2,6 SA receptors [29,65]. Furthermore, molecular markers, such as T105V and A661T, involved in adaptation to human-to-human transmission were identified in PB2 genes of all the strains [21]. These findings highlight that the Senegalese A/H9N2 AIV has the potential for cross-species transmission, as evidenced by the recent emergence of the first human case of A/H9N2 infection in Senegal [11]. The Senegalese human strain (A/Senegal/0243/2019) and the avian A/H9N2 viruses collected from poultry in the LBMs exhibited the same profile of human host adaptation markers. Although well-known substitutions in PB2, namely E627K and D701N, were not found in any of the Senegalese A/H9N2 strains, the presence of other mutations linked to increased polymerase activity, host specificity shifts, or increased virulence, such as PB2-A588V, PB1-H99Y, PA-V100I, PA-S409N, NP-E372D, suggests that these viruses continue to evolve for better adaptation to humans and mice. Hence, continuous monitoring of these viruses through systematic surveillance programs is warranted.

Influenza viruses have the potential to develop resistance to antiviral drugs through genetic mutations. Therefore, assessing the effectiveness of available antivirals is vital for combating zoonotic AIVs as part of influenza pandemic preparedness strategies. Indeed, the resistance of these viruses to adamantanes is conferred by well-defined mutations, including the S31N substitution in the transmembrane region of the M2 protein, which has been reported to confer resistance to M2-channel blockers [66]. In this study, all M2 proteins of Senegalese A/H9N2 isolates had the primary adamantine-resistance marker, S31N. However, it should be noted that adamantanes are not recommended for treating influenza due to the widespread resistance found in both seasonal and potentially zoonotic AIVs [60]. Although the key molecular markers commonly linked to oseltamivir and zanamivir resistance, namely H274Y and R292K [41,67], were absent in the NA proteins of A/H9N2 isolates, several mutations associated with varying levels of resistance to widely used antiviral drugs, including oseltamivir, zanamivir, and laninamivir were observed in these viruses. The PA-I38T mutation has been reported to confer resistance to the cap-dependent endonuclease inhibitor baloxavir marboxil [42]. In line with the findings of Suttie et al. in Cambodia [60], none of the Senegalese isolates harbored this molecular marker.

In addition to mutations, glycosylation at the antigenic sites of HA is another important mechanism used by viruses to evade antibody-mediated immunity. It has been established that N and O-linked glycosylation in HA protein can alter the attachment and antigenicity properties of IAVs [68]. This study identified seven potential N-linked glycosylation sites in the HA protein at positions 29, 82, 105, 141, 298, 305, and 492, which were identical to those found in A/H9N2 viruses isolated in Egypt [29]. However, unlike the findings of Sayes et al. [65], where avian A/H9N2 viruses with eight N-linked glycosylation sites in the NA protein were isolated, six putative N-glycosylation sites at amino acid positions 44, 61, 70, 146, 234, and 329 were predicted in the Senegalese A/H9N2 strains. This glycosylation pattern has been reported to facilitate the cleavage of NA by cellular proteases, thereby promoting the spread of infection [29]. Additionally, we found that A/H9N2 AIVs isolated during this active surveillance were primarily under neutral evolution or purifying selection pressure, a finding that has also been reported in Cambodia [60].

However, our findings should be interpreted with caution since the number of A/H9N2 isolates sequenced was very limited, and the active surveillance is limited to just two LBMs in Dakar and may not be representative of the entire country. Such limited analysis might overlook important viral variants or emerging strains circulating in other markets or regions, potentially leading to an incomplete understanding of the virus’s evolution and transmission dynamics. Therefore, additional investigations in more poultry markets and among wild birds in the country’s wetlands are necessary to provide a more comprehensive analysis of the epidemiology of AIVs in Senegal, ultimately aiding in better assessing their zoonotic potential.

## 5. Conclusions

In conclusion, this study provides an overview of the epidemiology and genetic characteristics of AIVs circulating within the poultry population in Senegal. Results from the active surveillance indicate high levels of AIV circulation in Senegalese LBMs, with the A/H9N2 subtype being the only influenza virus detected. Additionally, carcass wash water and poultry drinking water samples were most efficient at detecting the A/H9N2 virus.

Phylogenetic analysis revealed that the avian A/H9N2 viruses in this study belonged to the G1 lineage and were closely related to viruses identified in neighboring sub-Saharan countries, confirming the transboundary spread of these infections, possibly through cross-border movement of live poultry.

Furthermore, molecular analyses of the deduced amino acid sequences suggest that the Senegalese A/H9N2 strains may be adapted to mammalian species, with enhanced virulence, increased polymerase activity, and potential resistance to antiviral drugs, raising concerns about their zoonotic potential. However, further confirmation through animal studies is needed.

## Figures and Tables

**Table 1:** Positivity rate for avian influenza viruses in live bird markets in Senegal, December 2023–October 2024.

Type/Subtype	Cloacal Swab, No (%)	Poultry Feces, No (%)	Drinking Water, No (%)	Carcass Wash Water, No (%)	Total, No (%)
	N = 139	N = 128	N = 138	N = 94	N = 499
Influenza A	61 (43.9)	65 (50.8)	81 (58.7)	84 (89.4)	291 (58.3)
H9	18 (29.5)	27 (41.5)	46 (56.8)	33 (39.3)	124 (42.6)
H5	0 (0.0)	0 (0.0)	0 (0.0)	0 (0.0)	0 (0.0)
H7	0 (0.0)	0 (0.0)	0 (0.0)	0 (0.0)	0 (0.0)

**Table 2:** Description of the complete genomes of the H9N2 viruses obtained.

			PB2		PB1		PA		HA		NP		NA		MP		NS	
Sample ID	Sample Type	Location	Depth	% Coverage	Depth	% Coverage	Depth	% Coverage	Depth	% Coverage	Depth	% Coverage	Depth	% Coverage	Depth	% Coverage	Depth	% Coverage
GA/02/2023	Cloacal swab	Tilene	41.2	99.4	66.8	95.7	137.6	95.2	31.7	97.3	83.7	95	1.6	12.1	59.7	84.1	10.3	82
GA/08/2023	PDW	Thiaroye	379.4	100	472.2	100	67.1	79.8	1170	100	586.7	100	2191	100	7737	99.7	13,108	100
GA/13/2023	feces	Thiaroye	230.3	58.8	661.5	72.8	86.8	39.3	175.7	100	189	100	828	92.	10,241	99.9	31,387	100
GA/24/2023	CWW	Thiaroye	1033.4	95.8	511.3	100	436.7	99.6	1185.6	100	382.3	99.5	3054	100	11,593	99.7	14,350	100
GA/08/2024	PDW	Thiaroye	804.3	100	633.6	100	192.8	99.5	1252.5	100	890.8	100	2258	100	13,361	99.7	18,123	100
GA/46/2024	PDW	Thiaroye	375.7	100.0	571.7	100	142.3	84.5	1003.1	100	506.8	100	2336	100	12,504	99.8	15,386	97.6
GA/79/2024	CWW	Thiaroye	1321.3	100	655.2	100	375.7	99.9	2113.2	100	576.7	100	3699	100	13,925	99.7	23,532	100

PDW = Poultry drinking water, CWW = Carcass wash water.

**Table 3:** Amino acid substitutions associated with host specificity shift and virulence in the Senegalese H9N2 viruses.

Mutations in This Study	Equivalent Mutations in the Literature	Protein	Effects	Hosts	References
S121T	H103Y from series H103Y, T156A, Q222L, G224S		Host specificity shift	Ferrets	[23]
S143T	A131D		Antigenic drift	Human	[24]
S145T	L129V from series L129V, A134V		Host specificity shift	Avian, Human	[25]
A150S	A134V, S138A		Host specificity shift, Virulence	Swine, avian, Human	[26,27]
N167G	S159N	HA	Host specificity shift	Ferrets	[28]
A168N	T160A		Host specificity shift	Avian, Human	[28]
N191H	-		Host specificity shift	Avian	[29]
Q235I	S227N		Host specificity shift	Avian, Human	[30]
V327I	T381I from series N158D, N224K, Q226L, T381I		Host specificity shift	Ferrets	[31]
S353P	P15S		Increased virulence	Mice	[32]
T139N	T139A	M1	Increased virulence	Mice	[33]
T14M	M14Y		Reduced virulence	Mice	[34]
M100I	M100I	NS2	Reduced virulence	Human	[35]
E70A	K70E		Host specificity shift	Human	[36]
I81T	I81M		Host specificity shift	Avian, Human	[37]
I106M	M106I		Virulence	Human	[38]
S212P	P212A from series P212A, P215A		Reduced virulence	Human	[39]
V222M	C-terminus (RSEV)	NS1	Increased virulence	Mice	
E223A	C-terminus (RSEV)		Increased virulence	Mice	
I226V	C-terminus (RSEV)		Increased virulence	Mice	[40]
E227G	C-terminus (RSEV)		Increased virulence	Mice	
P228S	C-terminus (RSEV)		Increased virulence	Mice	

**Table 4:** Molecular characteristics of the hemagglutinin amino acid sequences in H9N2 viruses isolated in LBMs in Senegal.

Virus Name	RBS (H3 Numbering)				Cleavage Peptides	HA Predicted N-Glycosylation Sites (H9 Numbering)						
	I155T	S158N	T190V	Q226L		29–32	82–85	105–108	141–144	298–101	305–308	492–495
A/Senegal/0243/2019 *	T	S	R	L	RSSR/GLF	NSTE	NPSC	NGTC	NVTY	NSTM	NISK	NGTY
A/Chicken/Senegal/GA002/2023	T	S	M	L	RSSR/GLF	NSTE	NPSC	NGTC	NVTY	NSTM	NISK	NGTY
A/Environment/Senegal/GA008/2023	T	S	M	L	RSSR/GLF	NSTE	NPSC	NGTC	NVTY	NSTM	NISK	NGTY
A/Environment/Senegal/GA013/2023	T	S	M	L	RSSR/GLF	NSTE	NPSC	NGTC	NVTY	NSTM	NISK	NGTY
A/Environment/Senegal/GA024/2023	T	S	M	L	RSSR/GLF	NSTE	NPSC	NGTC	NVTY	NSTM	NISK	NGTY
A/Environment/Senegal/GA008/2024	T	S	M	L	RSSR/GLF	NSTE	NPSC	NGTC	NVTY	NSTM	NISK	NGTY
A/Environment/Senegal/GA046/2024	T	S	M	L	RSSR/GLF	NSTE	NPSC	NGTC	NVTY	NSTM	NISK	NGTY
A/Environment/Senegal/GA079/2024	T	S	M	L	RSSR/GLF	NSTE	NPSC	NGTC	NVTY	NSTM	NISK	NGTY

* H9N2 virus detected from human in Senegal; RBS = Receptor binding site.

## Data Availability

The A/H9N2 sequences generated in this study have been deposited in GISAID database under accession numbers EPI_ISL_19475615, EPI_ISL_19475616, EPI_ISL_19475617, EPI_ISL_19474871, EPI_ISL_19474872, EPI_ISL_19474873, EPI_ISL_19474874.

## Supplementary Materials

The following supporting information can be downloaded at https://www.mdpi.com/article/10.3390/v17010073/s1, Figures S1–S6: Maximum-likelihood phylogenetic trees based on complete nucleotide sequences of the PB2, PB1, PA, NP, M, and NS genes of Senegalese A/H9N2 viruses isolated from live bird markets.
