# Supplementary material for: Genetic and Molecular Characterization of Avian Influenza A(H9N2) Viruses from Live Bird Markets (LBM) in Senegal
Source: Viruses. 2025 Jan 8;17(1):73. doi: 10.3390/v17010073 (PMC11769557; doi:10.3390/v17010073)
Supplement: Supplementary file 1 [file viruses-17-00073-s001.zip › viruses-3354941-supplementary.pdf]

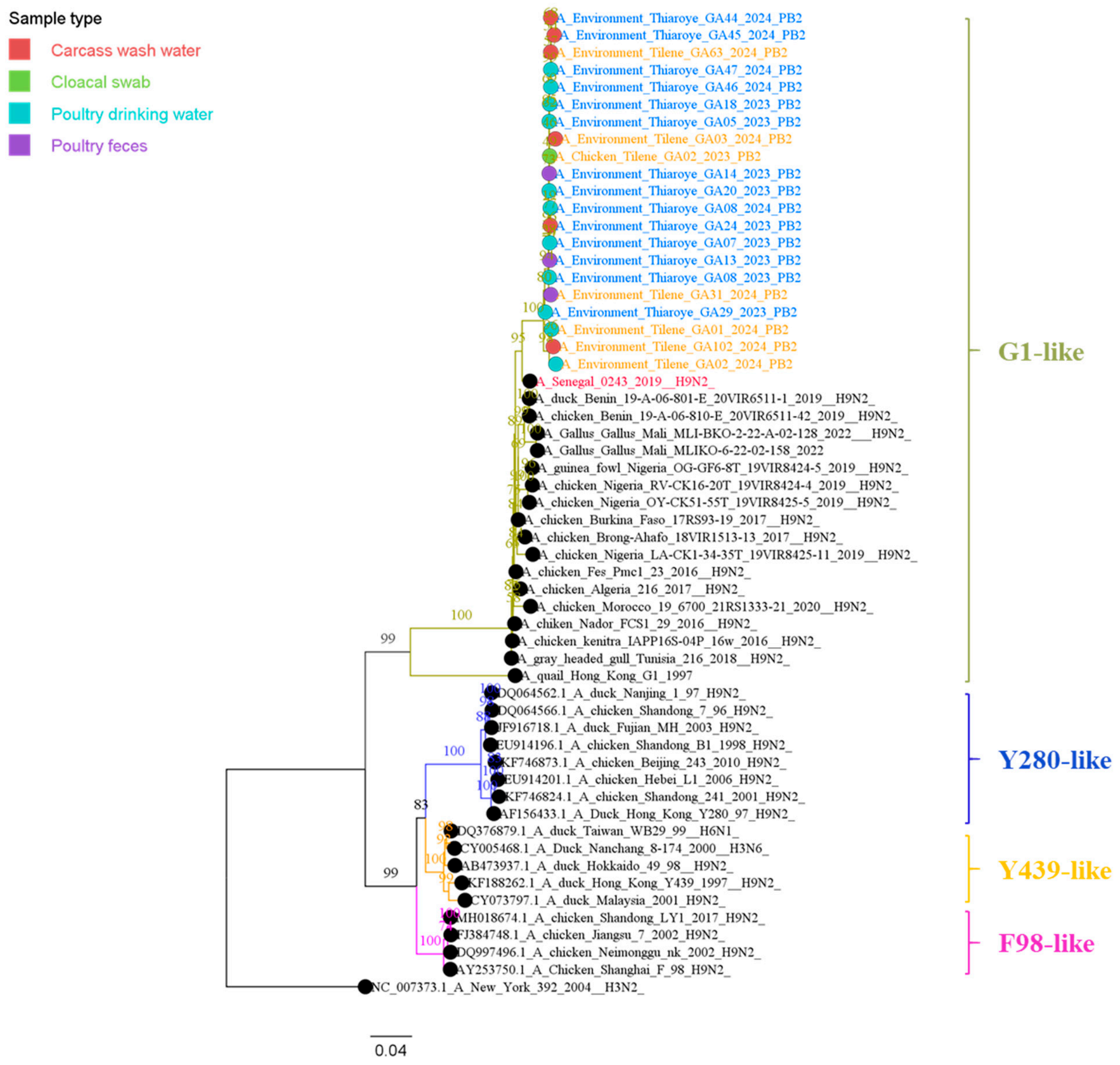

Figure S1. Maximum-likelihood phylogenetic tree based on complete nucleotide sequences of the **PB2** genes of Senegalese A/H9N2 viruses isolated from live bird markets (LBM). The tree was generated using IQ-TREE (v2.0.6) and visualized with FigTree (v1.4.4). Statistical significance was assessed using 1,000 bootstrap replicates, and the best-fit model was determined by the software. Sequences from Senegal are highlighted in blue for isolates obtained from the Thiaroye LBM, in orange for isolates from the Tilene LBM, and in red for the human A/H9N2 isolate.

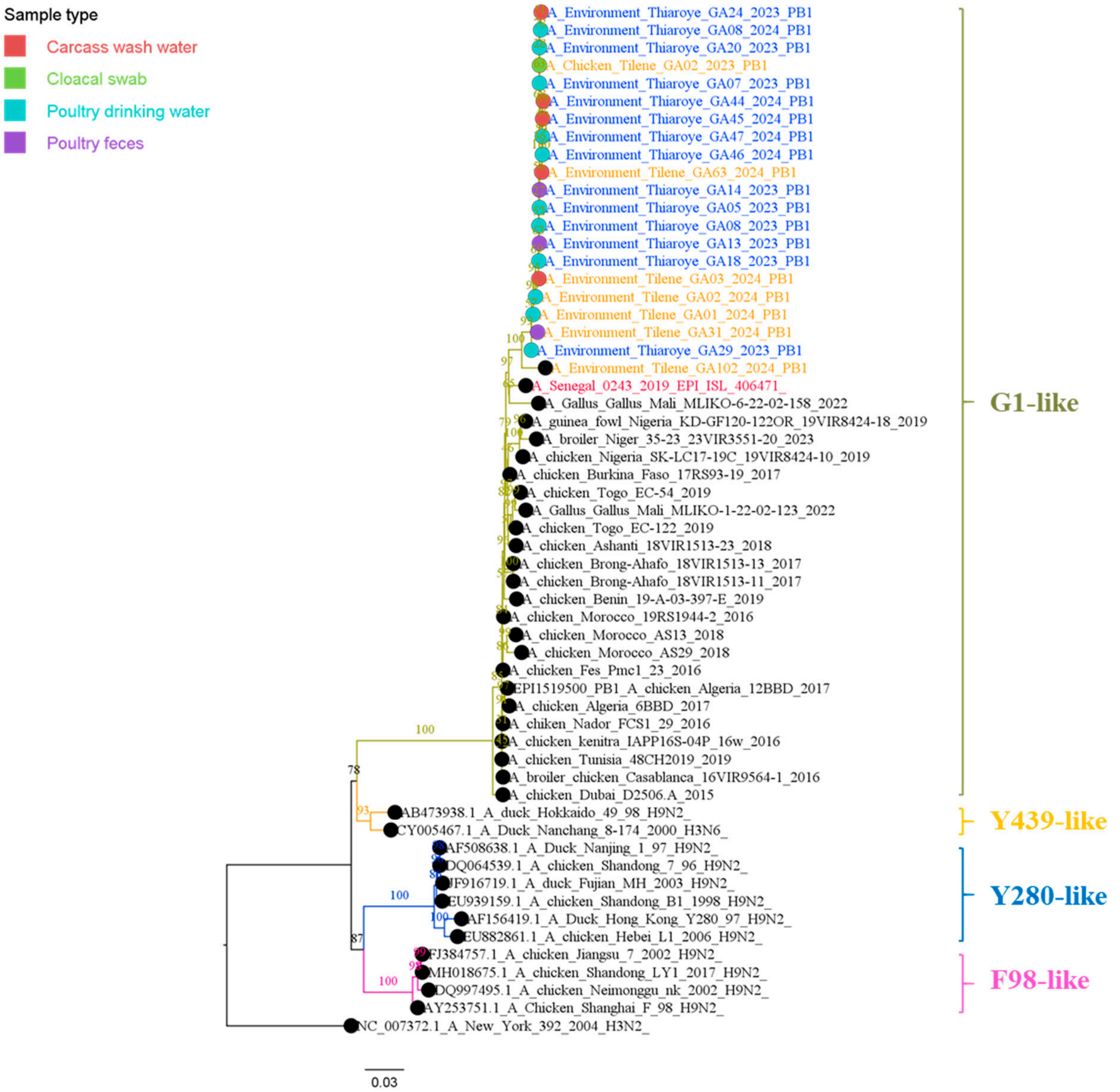

Figure S2. Maximum-likelihood phylogenetic tree based on complete nucleotide sequences of the **PB1** genes of Senegalese A/H9N2 viruses isolated from live bird markets (LBM). The tree was generated using IQ-TREE (v2.0.6) and visualized with FigTree (v1.4.4). Statistical significance was assessed using 1,000 bootstrap replicates, and the best-fit model was determined by the software. Sequences from Senegal are highlighted in blue for isolates obtained from the Thiaroye LBM, in orange for isolates from the Tilene LBM, and in red for the human A/H9N2 isolate.

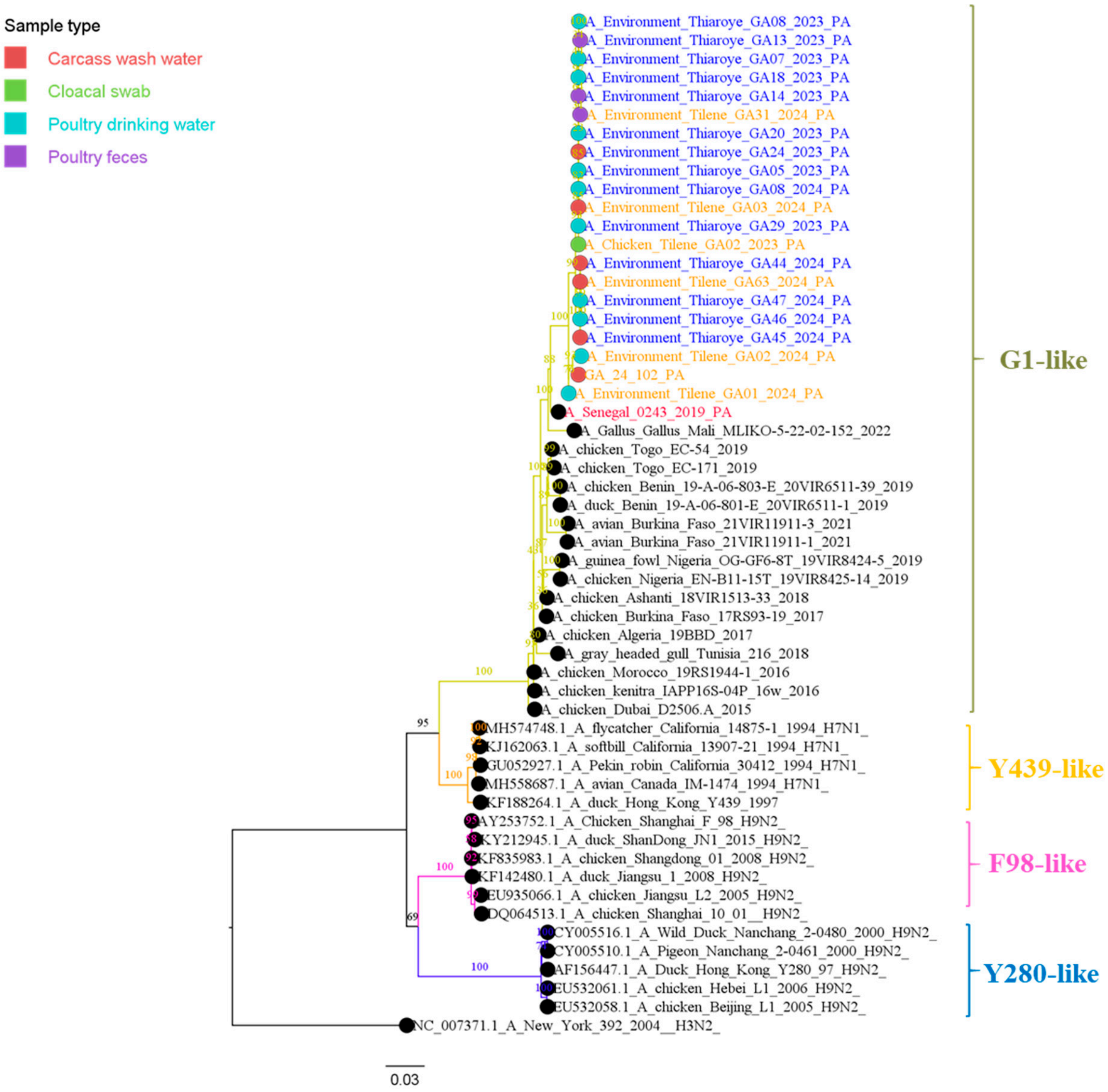

Figure S3. Maximum-likelihood phylogenetic tree based on complete nucleotide sequences of the **PA** genes of Senegalese A/H9N2 viruses isolated from live bird markets (LBM). The tree was generated using IQ-TREE (v2.0.6) and visualized with FigTree (v1.4.4). Statistical significance was assessed using 1,000 bootstrap replicates, and the best-fit model was determined by the software. Sequences from Senegal are highlighted in blue for isolates obtained from the Thiaroye LBM, in orange for isolates from the Tilene LBM, and in red for the human A/H9N2 isolate.

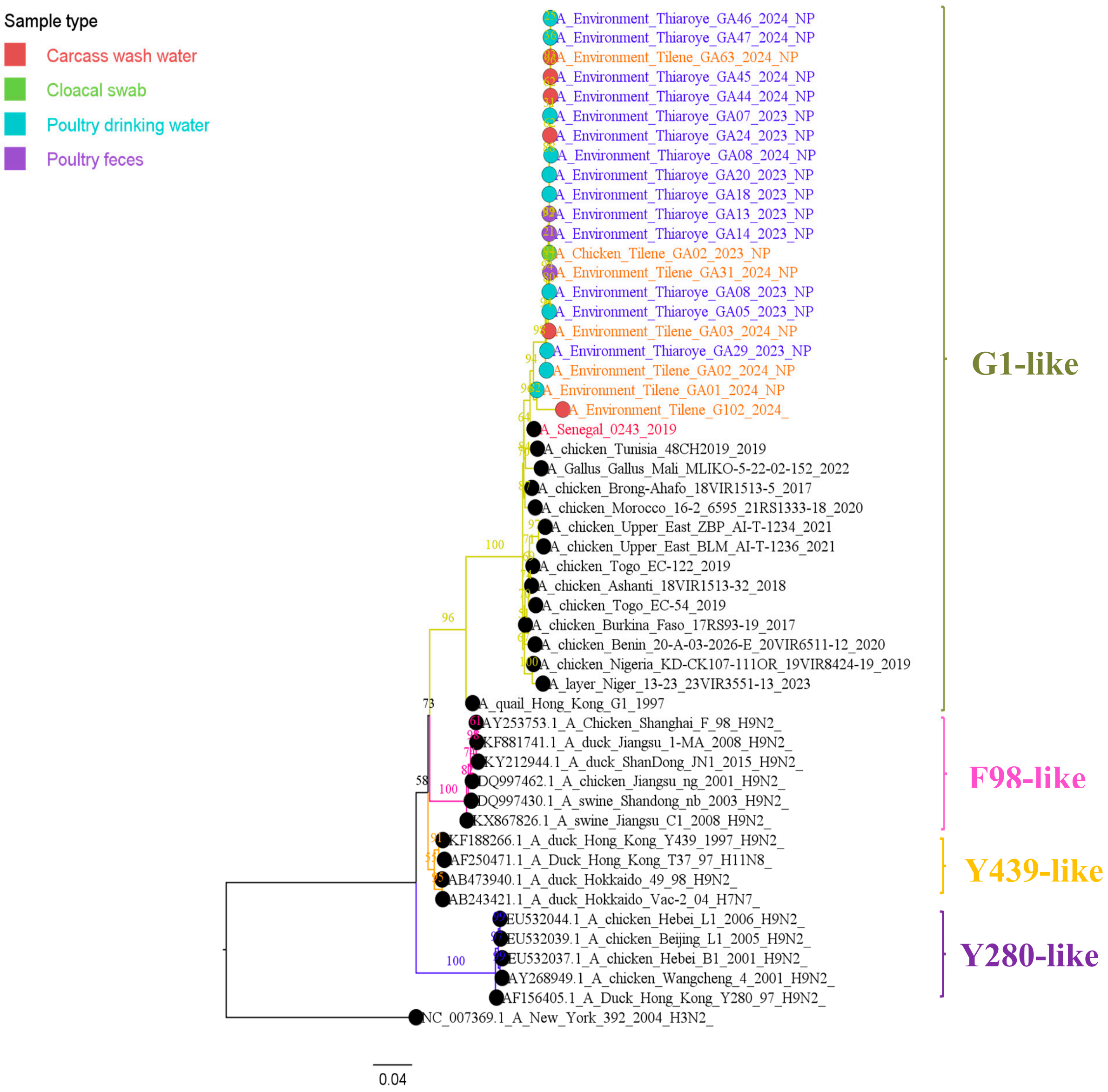

Figure S4. Maximum-likelihood phylogenetic tree based on complete nucleotide sequences of the **NP** genes of Senegalese A/H9N2 viruses isolated from live bird markets (LBM). The tree was generated using IQ-TREE (v2.0.6) and visualized with FigTree (v1.4.4). Statistical significance was assessed using 1,000 bootstrap replicates, and the best-fit model was determined by the software. Sequences from Senegal are highlighted in blue for isolates obtained from the Thiaroye LBM, in orange for isolates from the Tilene LBM, and in red for the human A/H9N2 isolate.

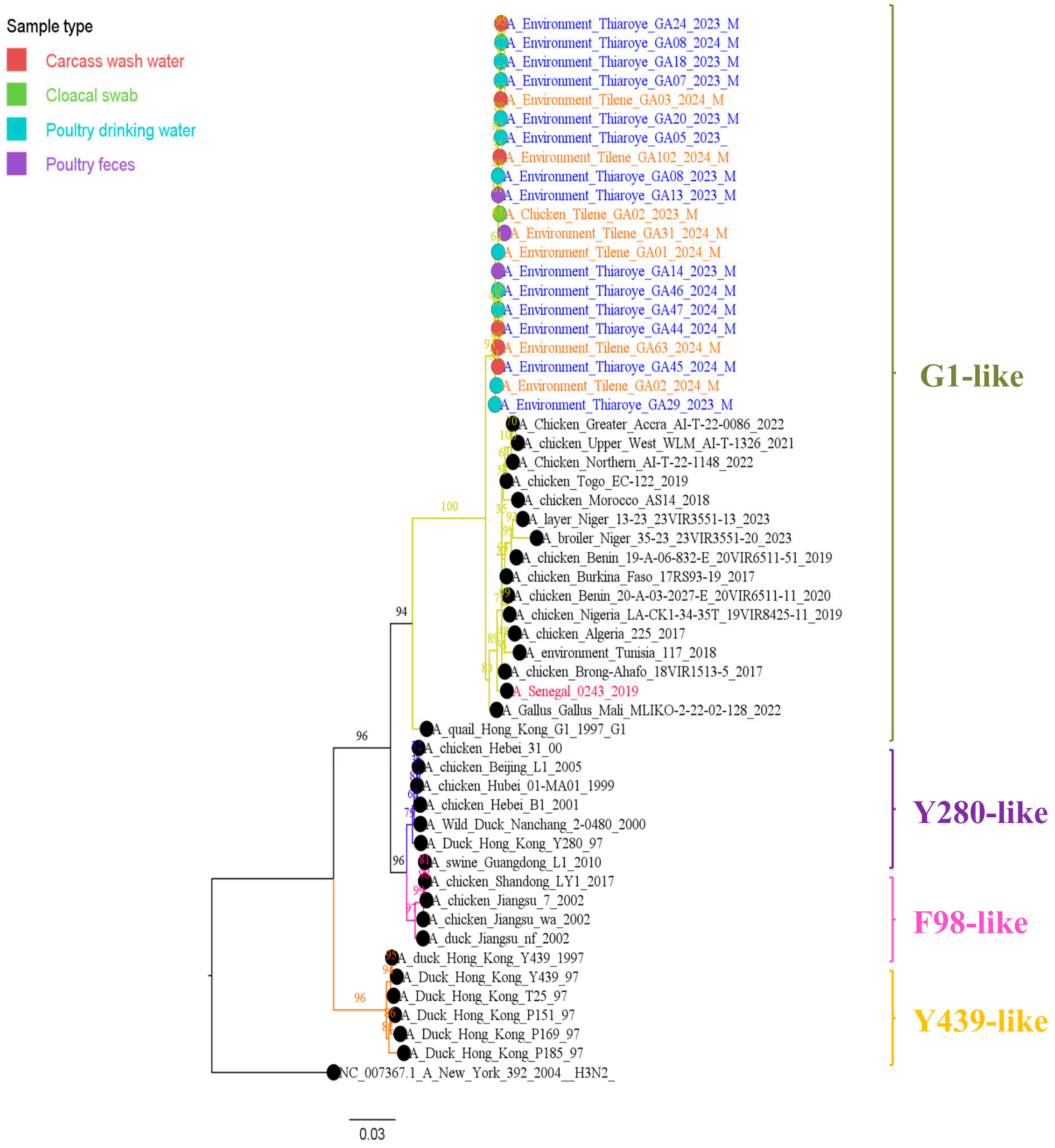

Figure S5. Maximum-likelihood phylogenetic tree based on complete nucleotide sequences of the **M** genes of Senegalese A/H9N2 viruses isolated from live bird markets (LBM). The tree was generated using IQ-TREE (v2.0.6) and visualized with FigTree (v1.4.4). Statistical significance was assessed using 1,000 bootstrap replicates, and the best-fit model was determined by the software. Sequences from Senegal are highlighted in blue for isolates obtained from the Thiaroye LBM, in orange for isolates from the Tilene LBM, and in red for the human A/H9N2 isolate.

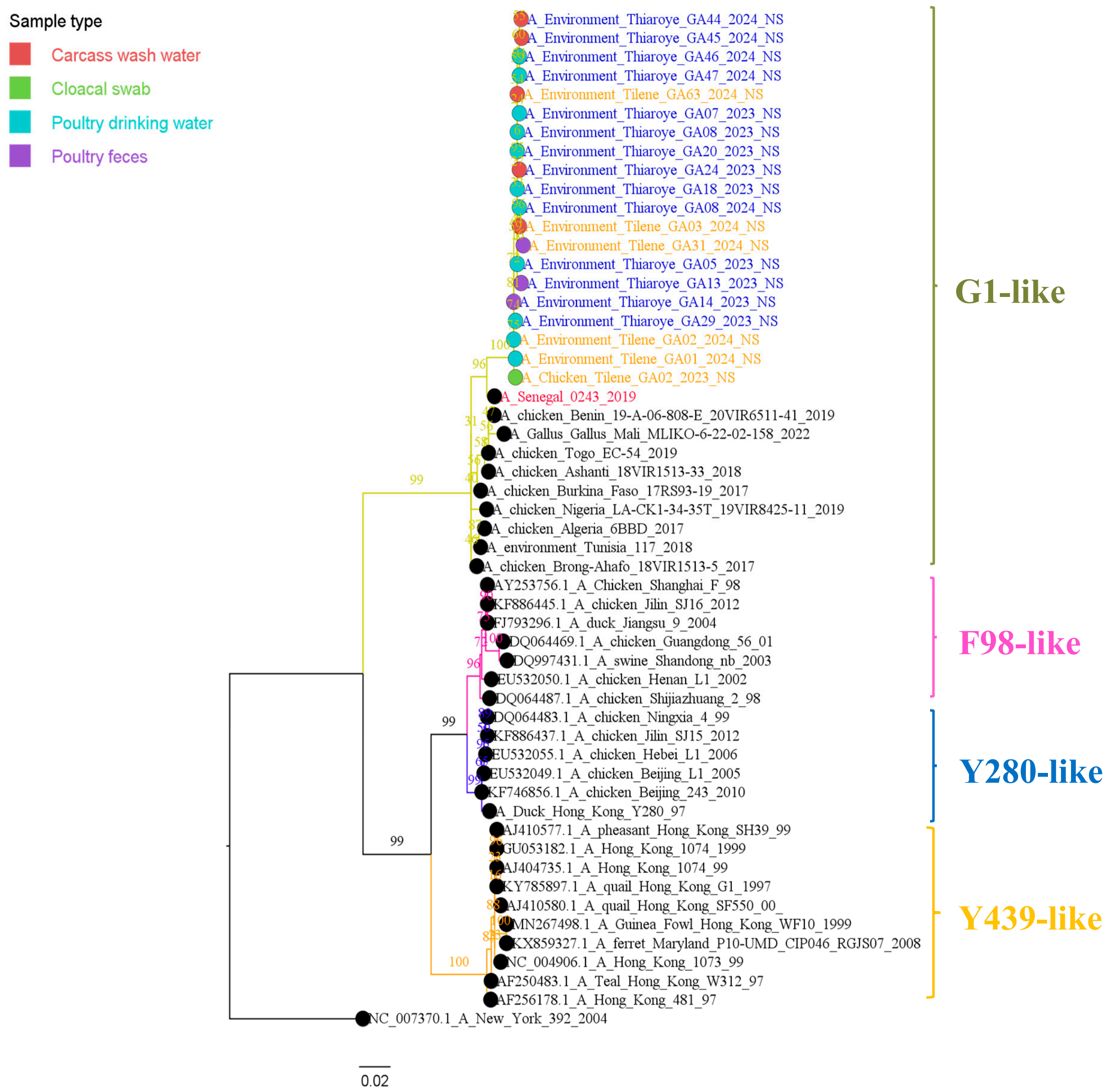

Figure S6. Maximum-likelihood phylogenetic tree based on complete nucleotide sequences of the **NS** genes of Senegalese A/H9N2 viruses isolated from live bird markets (LBM). The tree was generated using IQ-TREE (v2.0.6) and visualized with FigTree (v1.4.4). Statistical significance was assessed using 1,000 bootstrap replicates, and the best-fit model was determined by the software. Sequences from Senegal are highlighted in blue for isolates obtained from the Thiaroye LBM, in orange for isolates from the Tilene LBM, and in red for the human A/H9N2 isolate.
